# Exploring the Baseline Knowledge and Experience of Healthcare Professionals in the United Kingdom on Novel Psychoactive Substances

**DOI:** 10.3390/brainsci10030142

**Published:** 2020-03-02

**Authors:** Camille Ramos, Amira Guirguis, Nigel Smeeton, Hadar Zaman, Anna-Marie Felice, Stephanie Bancroft, Rosalind Gittins, Gill Hawksworth, John Martin Corkery, Fabrizio Schifano

**Affiliations:** 1 Psychopharmacology, Drug Misuse and Novel Psychoactive Substances Research Unit, School of Life and Medical Sciences, University of Hertfordshire, Hatfield AL10 9AB, UK; camille27ramos@gmail.com (C.R.); n.smeeton@herts.ac.uk (N.S.); j.corkery@herts.ac.uk (J.M.C.); f.schifano@herts.ac.uk (F.S.); 2 Swansea University Medical School, Institute of Life Sciences 2, Swansea University, Swansea SA2 8PP, UK; 3 Bradford School of Pharmacy & Medical Sciences, Faculty of Life Sciences, University of Bradford, Horton Road, Bradford BD7 1DP, UK; H.Zaman4@bradford.ac.uk; 4 CGL, St Martins House, 14 The Common, Hatfield AL10 0UR, UK; annamarie.felice@cgl.org.uk; 5 London North West Local Pharmacy Forum (LNWLPF) of the Royal Pharmaceutical Society, Pinner, Middlesex HA5 3EP, UK; steph_bancroft@hotmail.com; 6 Humankind, Inspiration House, Unit 22 Bowburn North Industrial Estate, Durham DH6 5PF, UK; rgittins1@nhs.net; 7 Royal Pharmaceutical Society, 66-68 E Smithfield, Whitechapel, London E1W 1AW, UK; gill.hawksworth@virgin.net

**Keywords:** novel psychoactive substances, NPS, healthcare professionals, drug education, substance misuse, harm reduction

## Abstract

**Objective**: This survey aimed to explore knowledge and experience on novel psychoactive substances (NPS) of healthcare professionals (HCPs). The study also aimed to assess how HCPs would like to improve their knowledge of NPS. **Methods**: Seventy paper questionnaires were disseminated in 2017 within continuing education events to pharmacists, nurses and general practitioners (GPs). Additionally, 127 online surveys were completed using the Qualtrics platform by other HCPs and mental health nurses in six United Kingdom (UK) independent mental health hospitals long-stay in-patient rehabilitation services. Two educational sessions involving pharmacists and GPs were also held in late 2017 and mid-2018. Knowledge of NPS by HCPs was evaluated prior to the start of the educational events. Evaluation forms were handed out post-sessions to garner feedback, especially on areas for improvement for future sessions. Statistical analysis of data was undertaken using SPSS (V.25). **Results**: Most HCPs reported only ‘basic’ to ‘intermediate’ NPS knowledge. Substance misuse service staff felt more informed, were more often consulted and had greater confidence regarding NPS compared to hospital and primary care professionals. A negative association was found between the age of the HCP and knowledge of NPS. Most participants expressed a need for regular training and updates as insufficient NPS-related information is currently received. **Conclusions**: An improvement within the self-reported knowledge of HCPs on NPS is evident in comparison to previous studies. Continued education of HCPs on NPS is fundamental for the provision of improved harm reduction services, which can enhance overall care for NPS service users.

## 1. Background

Numerous definitions of novel psychoactive substances (NPS) are currently used, most of which highlight their negative impact upon public health. The United Nations (UN) stated that NPS are “substances of abuse, either in a pure form or a preparation, that are not controlled by the 1961 Single Convention on Narcotic Drugs or the 1971 Convention on Psychotropic Substances, but which may pose a public health threat” [1]. The European Council’s definition places an emphasis on the threats posed by NPS and describes such a substance as “a new narcotic or psychotropic drug, in pure form or in preparation, that is not controlled by the United Nations drug conventions, but which may pose a public health threat comparable to that posed by substances listed in these conventions” [2]. The Advisory Council on the Misuse of Drugs (ACMD) in the United Kingdom (UK) uses a definition which incorporates both international and UK drug legislation: “Psychoactive drugs which are not prohibited by the United Nations Single Convention on Narcotic Drugs or by the Misuse of Drugs Act 1971, and which people in the UK are seeking for intoxicant use” [3]. The UK Psychoactive Substances Act (PSA) (2016) opted for a broader definition of NPS and defined them as “any substance, which is capable of producing a psychoactive effect” [4]. The term ‘new’ does not necessarily mean new inventions but instead addresses how such substances were recently made available, including failed pharmaceuticals or old patents which have been ‘rediscovered’ as ‘recreational’ molecules [5]. In addition, Corkery et al. [6] proposed that the term ‘new’ may correspond to an NPS molecule that has a ‘novelty’ appeal due to their innovative or unusual way of consumption. Ultimately, definitions of NPS vary according to the context(s) in which they are discussed [7]. 

Presently in the UK, NPS are regulated by the PSA 2016, which prohibits NPS production, sale, possession in custodial institutions or possession with intention to supply. This legislation introduced offences for the production, distribution, sale and supply of psychoactive substances in the UK for human consumption; it “builds on and complements” the Misuse of Drugs Act 1971 (MDA 1971) for the regulation of “dangerous or otherwise harmful” substances [4]. In accordance with the UK’s 2017 drug strategy, the PSA underwent a review by the Home Office in November 2018 to measure any changes in outcomes before and after the implementation of the Act [8]. The review showed that PSA enforcement led to the ban of NPS sale through headshops, has limited the sales and availability of NPS and reduced use, and as a result, there is evidence of reduction in health and social harms [8]. Deligianni and colleagues [9] evaluated the impact and effectiveness of the PSA 2016 through an updated survey in 2017 regarding NPS awareness, use, experiences and risk awareness and concluded that this regulation has not impacted on health risk awareness and motivations for NPS use in the UK.

The United Nations Office on Drugs and Crime (UNODC) classifies NPS into six categories: synthetic cannabinoid receptor agonists (SCRAs; e.g., AB-PINACA), stimulants (e.g., 4-fluoroamphetamine), classic hallucinogens (e.g., 2C-I), dissociatives (e.g., deschloroketamine), sedatives/hypnotics (e.g., etizolam) and opioids (e.g., furanyl fentanyl) [10]. Additionally, the ’plant-based’ category includes substances such as ‘khat’. 

From 2009 to December 2018, almost 900 different NPS from 119 countries and territories were reported to the UNODC Early Warning Advisory, most of which have been associated with unpredictable adverse effects [11]. According to the Global Drug Survey 2019, highly potent hallucinogen compounds including NBOMes and potent amphetamine analogues such as 4-fluoro-amphetamine have been associated with deaths in recent years and have remained increasingly problematic across Europe and Australia [12,13]. Furthermore, Poland ranked first as the country with the highest 12-month rates of NPS use by country globally, with England ranking seventh [13]. According to the Office for National Statistics (ONS) [14], a 29% reduction in the number of NPS-related poisoning deaths registered (as opposed to occurring) was recorded for both England and Wales following the introduction of the PSA 2016. However, the number of such events registered in 2018 showed a return to 2016 levels [14]. More importantly, the numbers of NPS-related poisoning deaths registered in Scotland have increased by 525% in the last four years, accounting for 45% of all drug-related poisoning deaths registered in 2018 [15]. Webb et al. [16] suggest that the total acute NPS toxicity presentations to a single emergency department (ED) in central London did not change significantly in the year after the introduction of the PSA 2016. Henshall et al. [17] recommended that government campaigns should cater for older individuals to help reduce the potential burden to the National Health Service arising from NPS-related hospital presentations. 

### 1.1. Current NPS Drug Scenes, Related Implications and Responses within the Healthcare System

The European Monitoring Centre for Drugs and Drug Addiction (EMCDDA) has advised that the proliferation of highly potent substances including new synthetic opioids (NSOs), for example, carfentanil, pose life-threatening poisoning risks which has overwhelmed law enforcement and healthcare systems [18]. Carfentanil is one of the most potent derivatives of fentanyl, being approximately 10,000 times more potent than morphine [19,20]. Limited research has been conducted to explore the toxicological profiles of newly emerging fentanyl derivatives, and this highlights the risks to humans who may be unaware of the presence of these active adulterants and other NSO when taking heroin or counterfeit pain medications [21]. Schifano et al. [22] identified 26,500 fentanyl misuse/abuse/dependence/withdrawal cases over 2004-2018 from pharmacovigilance databases including the European Medicines Agency (EMA), the UK Yellow Card Scheme and the United States Food and Drug Administration Adverse Event Reporting System. They recommended that fatalities associated with NSOs, especially fentanyls, should be thoroughly investigated using combined toxicological analyses. 

Evidence presented by the Home Office [8] suggests that the potency of SCRAs has increased despite the introduction of the PSA 2016. Martinotti et al. [23] reported that patients admitted to a psychiatric ward in a nightlife resort following the use of psychoactive substances, particularly those who reported recent use of SCRAs as their main substance, often presented with aggression on their arrival. Furthermore, violence during pre-admission and during admission to acute mental health services is more prevalent in NPS users compared to non-NPS users; thus, more education and support in the management of NPS users has been requested by HCPs who experienced an increasing risk of violence within their workplace [24]. Synthetic cathinones (SC) were the second most frequently seized group of NPS in 2016, where symptoms of intoxication can be long-lasting compared to SCRAs and may potentially be fatal [25,26]. Furthermore, SC such as mephedrone are commonly used during ‘chemsex’ [27,28]. Chemsex is associated with high-risk injection (known as ‘slamming’) and sexual risk-taking behaviour (e.g., unprotected sex), both of which may facilitate the transmission of sexually transmitted infections (STIs) or blood-borne viruses (BBVs) including hepatitis C virus (HCV) and human immunodeficiency virus (HIV) [29]. 

Diversion of prescription-only medicines (POMs) within an NPS context has been assessed by Chiappini and Schifano [30]. They evaluated the EMA’s EudraVigilance (EV) database on POM misuse-related adverse drug reactions (ADRs) over the last decade. A total of 7639 and 4301 ADR reports of misuse/abuse/dependence were respectively associated with the gabapentinoids pregabalin and gabapentin. They recommended that combined intake of opioids and sedatives leads to enhanced psychoactive effects, which has been shown to result in fatalities [31,32]. 

In the UK, stricter prescribing measures have possibly prompted the emergence of ‘designer’ benzodiazepines, which were not originally controlled drugs, such as the NPS phenazepam and etizolam [33,34,35]. Etizolam overtook diazepam as the benzodiazepine most frequently reported in drug-related deaths registered in Scotland in 2016 and again in 2017 [15,36,37]. The above shows the dramatic changes in the drug scene and the limited knowledge available to inform HCPs on adequate responses to these unknown substances.

### 1.2. Studies Exploring NPS Knowledge Among Healthcare Professionals (HCPs)

Previous studies have addressed the inadequacy in essential knowledge and skills required by HCPs on NPS to inform various interventions and highlighted the need for education on these novel molecules [38,39,40,41,42,43]. However, several studies only focused on specific professions and geographical locations in the UK [40,41,42,43]. The deficit in general knowledge on NPS may lead to inappropriate medical diagnoses and potentially the provision of inappropriate medical advice or interventions. Guirguis et al. [40] distributed a revised ‘ReDNet’ survey utilised initially by Simonato et al. [44]. This was an anonymous questionnaire that was part of a project funded by the European Commission to provide updates about NPS-related information [45,46]. London pharmacists in community settings were involved and most had poor knowledge of the 38 relatively well-known NPS listed in the survey, and few (*n* = 20; 37%) considered NPS as having ‘little’ work-related significance. Likewise, Campbell and colleagues [38] addressed how over 50% of HCPs did not have ‘adequate knowledge’ on drug interactions involving NPS, which is alarming and should be treated urgently through the delivery of appropriate training. Wood et al. [43] concluded that greater confidence was reported by both physicians and nurses on managing acute toxicity due to classical recreational drugs compared to NPS. Similarly, Owie et al. [42] found that most psychiatrists had inadequate overall knowledge of NPS. Lastly, Gittins et al. [39] utilised semi-structured interviews to gather detailed information from clients regarding their NPS use and concluded there is a need for further education and training for HCPs, who encounter NPS service users by adopting multi-agency and multi-disciplinary approaches to gauge awareness of changing trends in NPS use. When and where appropriate, referral to drug and alcohol treatment services or other professionals, such as psychiatry, sexual health or social services can be offered to individuals [47]. Additionally, Tracy and colleagues [48] listed the different NPS-related resources that are accessible to HCPs, including the UK National Poisons Information Service and its clinical toxicology database TOXBASE, NEPTUNE (novel psychoactive treatment UK network) and an NPS resource pack for informal educators and frontline practitioners. None of the above studies indicated an effective educational tool or strategy for HCPs on NPS.

Against the ever-changing backdrop of NPS, in the UK and elsewhere, and the demonstrable lack of awareness and need for education of HCPs concerning the phenomenon and how they can deal with the consequences of NPS use, the main aim of our study was to scope the perceptions of HCPs from various sectors and regions within the UK of these issues. Although the authors are cautious about the generalisability of the findings, the results provide an insight about the self-reported baseline knowledge of HCPs on NPS. This work aimed to build on what was already known with the inclusion of a wider range of HCPs from different health sectors. It is hoped that the findings may seek the views of HCPs on improvements required for education/training and public health intervention research by reviewing existing knowledge of NPS use.

## 2. Methods

### 2.1. Survey Design

**Figure 1:**
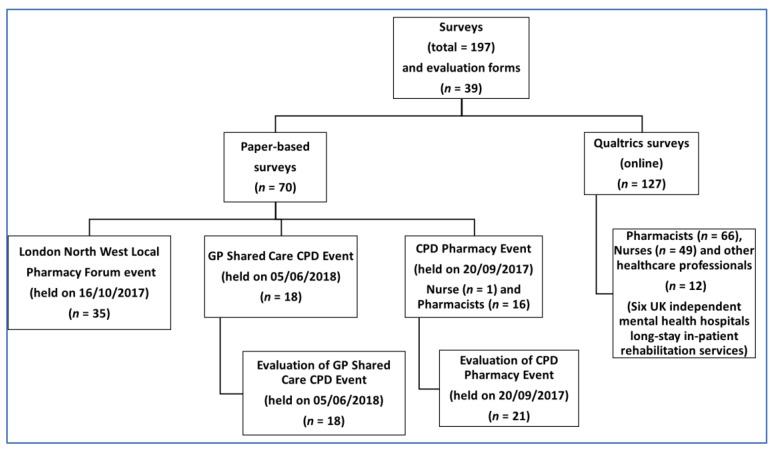
Survey distribution flow chart.

Both online and paper-copy surveys were utilised (Figure 1). The survey consisted of 31 questions, adapted from the ‘ReDNet’ survey, used initially by Simonato et al. [44], which was revised by Guirguis et al. [40], and updated with more recent NPS compounds. The survey questions included respondents’ demographic information (gender, age, health sector, profession, working town and country). Participants were then asked if they have heard of 93 compounds under six categories: POMs (*n* = 16), benzodiazepines (*n* = 8), SCRAs (*n* = 21), opioids (*n* = 16), SC (*n* = 17) and ‘others’ (*n* = 15). Participants could respond with a “yes”, “no” or “know of customers/patients who have abused it” for each compound.

The choice of NPS compounds was based upon formal reporting to the EMCDDA by its early warning system on NPS and lists contained in its European Database on New Drugs (EDND) [49]. NPSs included in the survey were associated with the highest number of fatalities prior to data collection. 

Likert scales were also used in the survey, including one where respondents were asked to rate their level of knowledge about NPS/’legal highs’, with 1 being “poor” and 5 being “very good”. Such scales are easier to read and complete, and offer a range of opinions that respondents can choose from [50]. Lastly, open-ended questions were utilised in the evaluation forms of the educational workshops, such as listing up to three aspects that were learnt from the session as well as a dedicated section for suggestions or any additional comments. This was advantageous as improvements for future sessions could be sought from respondents and gaps in knowledge about commonly abused/misused drugs could be identified. 

### 2.2. Survey Distribution

There were no pilot studies for this survey as it was adapted with a few minor amendments, such as updating the list of NPS (Table S1), from the same survey instrument used by Guirguis et al. [40]. The study targeted all pharmacists in the UK who are members of both the Royal Pharmaceutical Society (RPS) and the College of Mental Health Pharmacy (CMHP), as well as other healthcare professionals within substance misuse and mental health services. Purposive sampling was implemented, which involved adult HCPs (18 years and older) completing paper-based surveys distributed by the individuals leading the educational events for pharmacists, nurses and general practitioners (GPs). 

As illustrated in Figure 1, the online survey (File S1) was delivered via the Qualtrics platform (https://www.qualtrics.com/). Survey links were sent to mental health nurses working across six independent mental health hospitals (non-National Health Service (NHS) hospitals) to mental health pharmacy professionals via CMHP. Likewise, the RPS Science educational lead sent links to all RPS London local practices for community pharmacists. Responses were received by Qualtrics up to 15 October 2017.

Leaders in charge of the LNWLPF event on 16 October 2017 and the ‘Change, Grow, Live’ (CGL) charity events disseminated paper-based surveys on premises. Lastly, at the end of each of the two educational workshops (both for the Pharmacy and GP Shared Care Continuing Professional Development (CPD) events), feedback forms were provided.

### 2.3. Data Extraction, Cleaning and Analysis

Statistical analysis for both datasets from paper-based and online surveys was carried out using the statistics software package SPSS V.25 (IBM Corporation, Armonk, NY, USA). The single continuous variable—age—was checked for normality. Basic summary statistics were obtained as means or percentages. Qualitative variables were analysed using the chi-squared test, with the exact *p*-value being reported for contingency tables having more than 20% of cells with an expected value of less than 5. Where appropriate, Spearman’s correlation coefficient was used to assess associations for ordered and continuous variables. Text from open-ended questions was analysed according to emerging themes. 

## 3. Results

### 3.1. Participants

**Figure 2:**
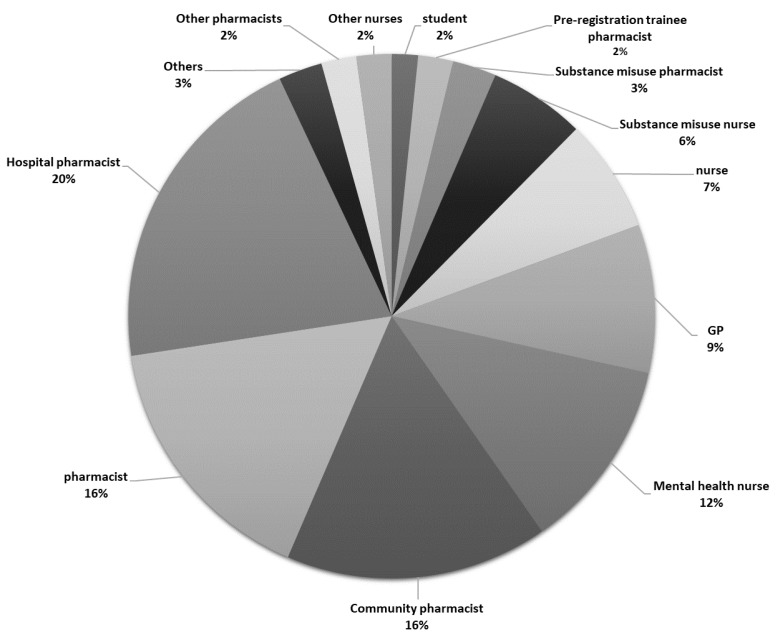
Profession types (*n* = 186).

**Figure 3:**
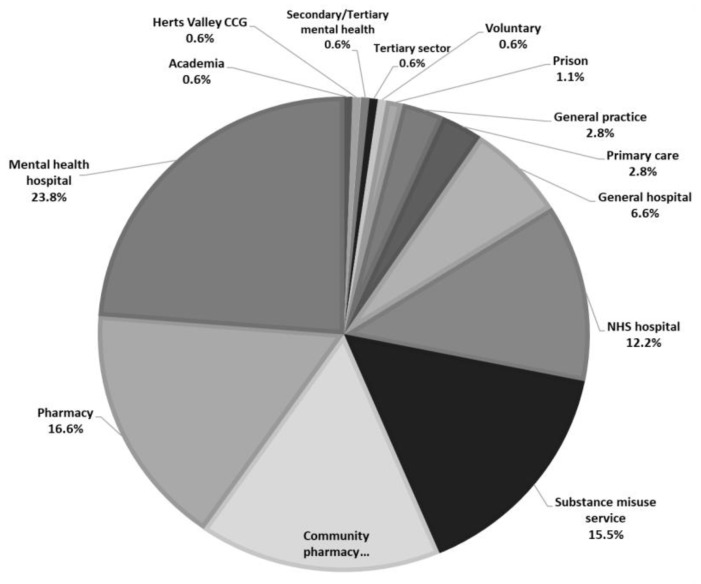
Health sectors (*n* = 181).

There was sufficient data from 197 responses for inclusion in the analyses: 127 (64.5%) respondents fully completed the online surveys and 70 (35.5%) paper-based surveys were fully completed. Forty-four of the online responses could not be used (e.g., partially completed). The majority of respondents were females (*n* = 138; 70.4%). Age had an approximate normal distribution with a mean of 41.79 years (*SD* = 12.4). Most respondents qualified between the years 2000-2009 (*n* = 51; 27.1%) and 2010 and later (*n* = 58; 30.9%). The mean number of years qualified was 17.66 ± 12.1 (range: 1-48) years. Most respondents were from England (*n* = 183; 95.9%), with four (2.1%) from Wales and four (2.1%) from outside the UK. The most represented Government Office Region was the North (n = 55; 28.8%), followed by Greater London and the South East (*n* = 49; 25.7%), the East of England (*n* = 45; 23.6%), the Midlands (*n* = 19; 9.9%), and the South West (*n* = 15; 7.9%). From the respondents who stated their profession (*n* = 186), the largest group of HCPs were pharmacists (*n* = 107; 57.5%), of whom 30 were community pharmacists (16.1%), 38 (20.4%) were from hospitals and 5 (2.7%) worked in substance misuse services. Additionally, 50 nurses (26.9%) were involved, where 22 (11.8%) had a mental health background, 11 (5.9%) worked in a substance misuse setting, 2 (1.1%) were non-medical prescribers and 15 (8.1%) belonged to other health sectors. General practitioners comprised the third largest group of HCPs (*n* = 17; 9.1%). Figure 2 and Figure 3 provide further specifics relating to professions and health sectors.

Also includes other pharmacists (advanced specialist pharmacist, prison pharmacist, clinical pharmacist, general practitioner (GP) pharmacist), other nurses (learning disabilities nurse, drug and alcohol liaison nurse, non-medical prescriber nurses) and others (family safeguard worker, substance misuse manager, pharmacy technician, university lecturer).

### 3.2. Knowledge of NPS

**Figure 4:**
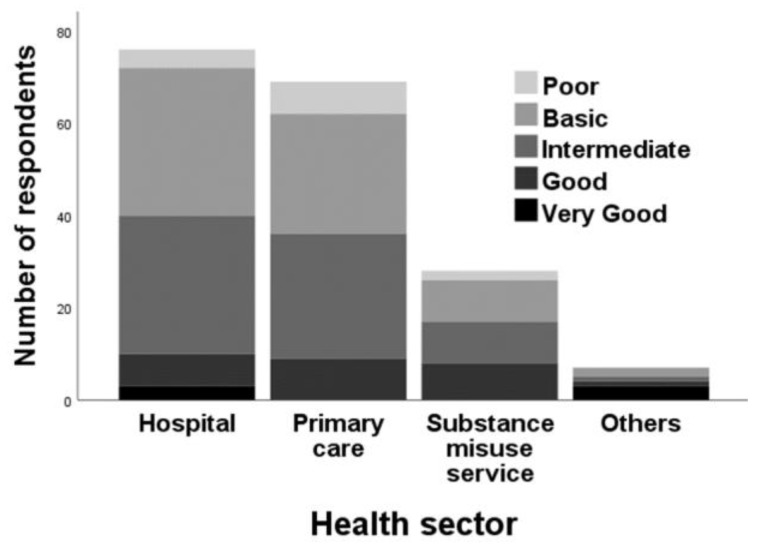
Self-reported level of knowledge on novel psychoactive substances (NPS) among different health sectors.

When asked about their expertise/knowledge of NPS (Figure 4), most perceived themselves as having a ‘basic’ (*n* = 76; 39.2%) or an ‘intermediate’ level (*n* = 73; 37.6%) of knowledge. By contrast, 14 HCPs (7.2%) regarded themselves as having ‘poor’ NPS knowledge, followed by ‘good’ (*n* = 25; 12.9%) and ‘very good’ (*n* = 6; 3.1%). A negative association was found between the age of the HCP and knowledge of NPS (Spearman’s correlation = − 0.176, *p* = 0.015).

For the 16 listed POMs, zopiclone was the most recognised (*n* = 109; 64.1%), followed by gabapentin (*n* = 106; 62.7%) and pregabalin (*n* = 103; 60.2%). The top three POMs that HCPs claimed to be misused by customers/patients were pregabalin (*n* = 48; 28.1%), zopiclone (*n* = 47; 27.6%) and gabapentin (*n* = 41; 24.3%). Of the opioids (*n* = 16) included, carfentanil (*n* = 41; 29.3%) was the most recognised, followed by benzoyl fentanyl (*n* = 39; 28.3%), furanyl fentanyl (*n* = 32; 23.4%) and then 3-fluorofentanyl (*n* = 31; 22.6%). Six (4.4%) claimed that 3-fluorofentanyl was used by customers/patients. 

Of the 17 listed SC, mephedrone (*n* = 62; 44.9%) was the most recognised, where 15 HCPs (10.9%) were also aware of its use by customers/patients. The most commonly known benzodiazepines (*n* = 8) were 4-chlorodizepam (*n* = 46; 32.9%), phenazepam (*n* = 19; 13.8%) and bromazolam (*n* = 18; 13.2%). 

**Figure 5:**
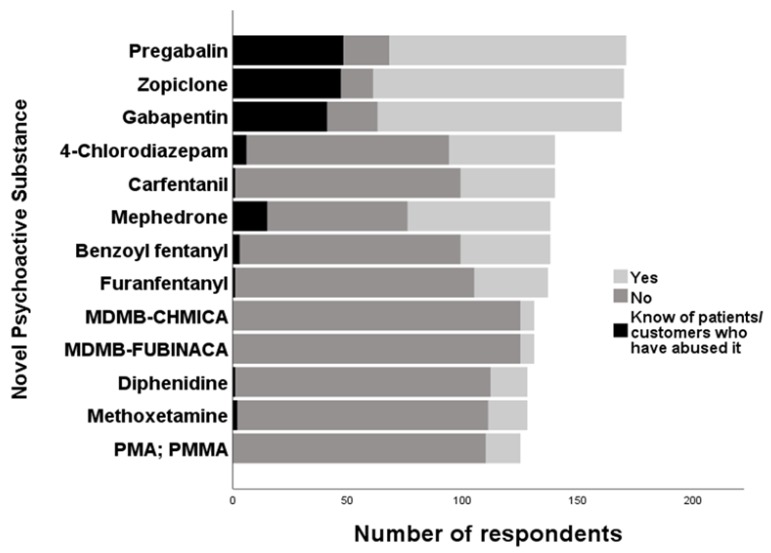
Most commonly known NPS by healthcare professionals (HCPs).

For the 21 listed SCRAs, MDMB-CHMICA (*n =* 6; 4.6%) and MDMB-FUBINACA (*n* = 6, 4.6%) were the most commonly known. Lastly, for NPS that were classified as ‘others’ (*n* = 15), the most recognised compounds included methoxetamine (*n* = 17; 13.3%), diphenidine (*n* = 16; 12.5%) and PMA/PMMA (*n* = 15; 12.0%). The most commonly known NPS are shown in Figure 5.

Several respondents also stated the names of the NPS they were aware of. ‘Spice’ was mentioned 26 times (53.1%), followed by ‘Black Mamba’ (*n* = 4; 8.2%), ‘synthetic cannabinoids’ (*n* = 4; 8.2%), ‘poppers’ (*n* = 2; 4.1%) and even ‘vapes’ (*n* = 1; 2.0%) were stated.

In terms of NPS route of administration, 155 HCPs (78.7%) were aware of oral, followed by smoking (*n* = 148; 75.1%), inhaling (*n* = 136; 69.0%), snorting (*n* = 131; 66.5%) and through the vaginal route (*n* = 28; 14.2%). Other administration routes include vaping (*n* = 94; 47.7%), ‘bombing’ (*n* = 61; 31.0%), rectal administration (*n* = 47; 23.9%) and ‘slamming’ (*n* = 27; 13.7%). ‘Bombing’ involves grounding and wrapping the drug in a cigarette paper then swallowed, whereas ‘slamming’ is the injection of psychostimulants (e.g., mephedrone) in a sexual context to improve mood and facilitate sexual disinhibition [51,52].

Of those HCPs who encountered customer/patients seeking advice about the management of adverse effects from NPS (*n* = 49), 18.4% were nurses (*n* = 9) and 20.4% (*n* = 10) were hospital pharmacists. Likewise, 22.4% (*n* = 11) were nurses with similar encounters in substance misuse services. Eighty-three HCPs claimed to have counselled NPS/‘legal highs’ users, where HCPs from hospital settings including nurses (*n* = 19; 22.9%), pharmacists (*n* = 14; 16.9%) and GPs (*n* = 3; 3.6%) were involved. GPs in primary care also provided counselling (*n* = 5; 6.0%). The majority of those who provided counselling in substance misuse settings were nurses (*n* = 17; 20.5%). In contrast, the HCPs who did not counsel customers/patients who use NPS (*n* = 78) were mainly pharmacists in primary care (*n* = 29; 37.2%). Counselling may involve the provision of advice on the safe management of adverse effects or about the safe use of NPS (i.e., safer injecting techniques or through needle and syringe exchange programs). However, the authors could not identify from the collected data whether the counselling was simple advice, or part of a brief intervention or structured counselling.

Moreover, participants were then asked to elaborate any ADRs they had encountered, resulting from NPS use. Physical- and mental health-related symptoms were frequently experienced by NPS service users, where hallucinations and paranoia were commonly reported. Most HCPs were ‘not confident’ in providing advice about safe use of NPS/’legal highs’ (*n* = 97; 50.3%). Furthermore, most HCPs (*n* = 109; 60.6%) assessed themselves as ‘not confident’ about the provision of advice regarding the safe management of ADRs resulting from NPS use. Among the 43 HCPs who were ‘fairly confident’ on this aspect, 16 were from hospitals (37.2%), whilst 14 were from substance misuse services (32.6%).

### 3.3. Relevance of NPS to HCPs

Grouping respondents by health sector (Table 1) and profession (Table 2) demonstrated important differences. Reported knowledge regarding NPS was somewhat greater for nurses compared to GPs and pharmacists. Nurses were more likely to be asked for advice regarding NPS use and felt more knowledgeable and confident about giving such advice.

Substance misuse service staff reported greater knowledge regarding NPS relative to hospital and primary care professionals. Substance misuse service professionals were also more likely to receive requests for advice and counselling regarding NPS and felt more confident about giving advice than professionals working in hospitals or primary care.

Most respondents believed that having knowledge about NPS is ‘fairly’ significant (*n* = 69; 41.8%) for their work. However, many were unaware of websites or videos promoting NPS use (*n* = 150; 84.7%). Although 120 HCPs (68.2%) were aware of illegitimate online ‘pharmacies’ selling medicinal products, most of the respondents were unaware of the existence of Report Illicit Drug Reactions (RIDR), the Public Health England/MHRA reporting system (https://report-illicit-drug-reaction.phe.gov.uk/) for NPS (*n* = 134; 73.2%). The RIDR database officially closed down in January 2020 due to lack of reporting by HCPs. Moreover, 118 HCPs (67.0%) claimed a lack of awareness about NPS use for improvement of physical/mental performance. Additionally, the average age range of customers/patients using NPS was 20-29 years old, as claimed by 108 HCPs (65.9%). 

Furthermore, 164 HCPs (92.1%) stated that they do not receive enough information about NPS to safely care for their patients. As indicated in Table 3, several HCPs mentioned the invaluable impact of education on NPS to keep pace with the constantly changing drug scenes. Nonetheless, most received NPS information from colleagues (*n* = 100; 50.8%), media (e.g., TV; *n* = 99; 50.3%), specific websites (e.g., www.novelpsychoactivesubstances.com; *n* = 73; 37.1%), followed by email (*n* = 59; 29.9%), service users (*n* = 57; 28.9%), scientific literature (*n* = 56; 28.4%), other websites (e.g., for a; *n* = 48; 24.4%), conferences/seminars/workshops (*n* = 44; 22.3%), online courses (*n* = 25; 12.7%), others (*n* = 12; 6.1 %) and short message services (SMS; *n* = 5; 2.5%).

Lastly, some participants also emphasised the growing complexity surrounding NPS, which “*still has elements of being unclear of what is legal*” (nurse, mental health hospital). Perhaps, the potency of such substances may be increased as they “get more designer and specific”; thus, requiring a basic understanding on NPS is crucial to “*formulate a baseline approach as names and formulas change*” (pharmacist, substance misuse service). Several HCPs mentioned the invaluable impact of education on NPS to keep pace with the constantly changing drug scenes. Consequently, it has been addressed how education on NPS should be introduced early on and “be taught in schools about their dangers” (general practitioner, general practice). A sense of urgency was highlighted to “*implement training as soon as possible…to keep abreast of information and dangers on current legal highs*” (family safeguard worker, substance misuse service). Lastly, the increasing pressure that drug treatment and recovery services experience was mentioned, who appear to be “*overworked*” (pharmacist, community). 

### 3.4. Feedback from Educational Training and Future Learning Needs

**Figure 6:**
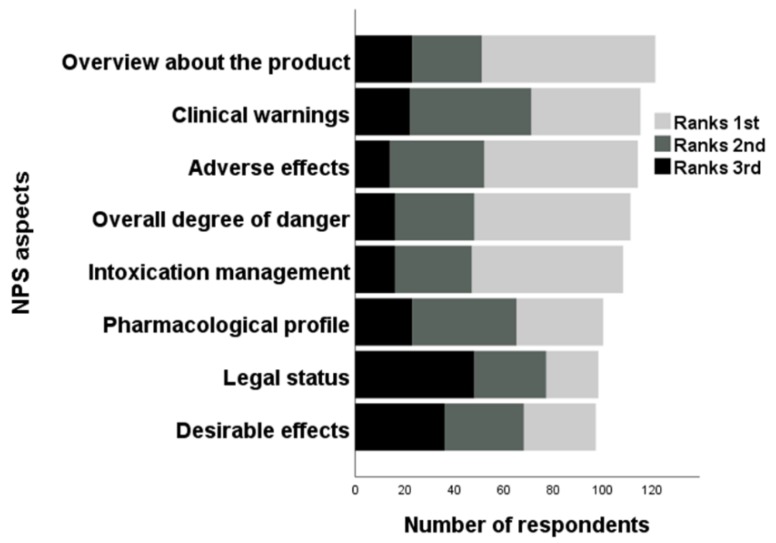
Ranking of various NPS aspects fundamental for improvement.

Of the 175 participants who gave information regarding their CPD in the previous 12 months, 152 (86.9%) included NPS/’legal highs’ as part of their training and 167 (95.4%) believed that NPS should be integrated into undergraduate curricula. When HCPs were asked about their desired area for improvement (Figure 6), ‘overview about product’ was most frequently ranked first (*n* = 70; 35.5%), ‘clinical warnings’ was most frequently ranked second (*n* = 49, 24.9%) and ‘legal status’ was most frequently ranked third (*n* = 48, 24.4%). Regarding available updates on NPS, 155 HCPs wanted to be informed regularly. Of these participants, preferred types of communication were emails (*n* = 136; 87.7%), online course (*n* = 77, 49.7%), specific websites (*n* = 75; 48.4%), followed by social networks (e.g., Facebook, Twitter; *n* = 19; 12.3%), SMS (*n* = 10; 6.5%) and ‘others’ (e.g., alcohol/drug misuse training; *n* = 8; 5.2%).

There were 168 HCPs who were willing to partake in further training on NPS. Preferences for mode of delivery were online (*n* = 108; 64.3%), via workshops (*n* = 99; 58.9%) and lectures (*n* = 99; 58.9%), followed by blended learning (*n* = 46; 27.4%), webinars (*n* = 42; 25.0%) and ‘others’ (e.g., written information sent to practices; *n* = 2; 1.0%).

Both the Pharmacy and GP Shared Care CPD Events addressed work-related premature mortality/intoxications related to NPS use (multi-drug use incorporating NPS) in different user groups. Feedback was obtained from the attendees (*n* = 18) of the GP Shared Care Event (Table 4). Most believed that the issue of NPS was ‘most certainly’ relevant to their work area (*n* = 12; 66.7%). Similarly, 12 GPs stated that it was ‘most certainly’ relevant to be aware of the risks of pregabalin and gabapentin in their daily work. There was an equal proportion of GPs that believed the event was either ‘excellent’ (*n* = 9; 50.0%) or ‘good’ (*n* = 9; 50.0%). Many GPs valued the topics discussed particularly about the pharmacokinetic and pharmacodynamic pathways of commonly abused drugs (Table 5). The lack of awareness amongst many GPs about the misuse potential of many POMs (e.g., pregabalin and fentanyl) was also highlighted (Table 6). Likewise, the CPD Pharmacy Event collected feedback from 21 attendees; the overall rating of the event was considered as ‘excellent’ by the majority (*n* = 20; 95.2%) (Table 5). Lastly, several participants from both events (Table 6) addressed the importance of being updated regularly about drugs of abuse and addiction in general.

## 4. Discussion

### 4.1. Characteristics of HCPs

Previously, Schifano et al. [53] stressed the need for HCPs to be continuously updated with regards to the constantly changing drug scenarios to identify new, emerging trends that can help with the provision of immediate interventions. Results (Figure 4) suggested improvements where self-reported levels of knowledge on NPS were mostly ‘basic’ (*n* = 76; 39.2%) and ‘intermediate’ (*n* = 73; 37.6%), which is in contrast to other studies that have reported inadequate HCP knowledge [40,41,42]. Nurses and pharmacists, predominantly working in hospitals and substance misuse services, had the most encounters with NPS use. Hence, this is an intervention opportunity to make individuals more aware of the health-related consequences of using drugs with misuse potential when they access hospitals or substance misuse services. Provision of counselling about NPS use was less frequently done, as claimed by pharmacists in primary care who completed our survey. However, this must be treated with some caution given that purposive sampling was utilised, which comprised a small group of HCPs and therefore cannot be representative of the above-mentioned cohort of HCPs in the UK. Lack of knowledge about NPS can lead to misdiagnoses and under-recognition of potential physical and psychological repercussions of NPS use; stigma attached to seeking help for substance misuse may lead to non-engagement by NPS service users [42,54]. Hence, the topics for future educational sessions should be tailored according to the type of HCP and/or their role(s) as the context or their work area, as well as the type of clientele, may have an influence on the type of response/intervention that can be conducted by HCPs. 

Both negative associations between age and ability to use electronic tools, and between age and level of NPS knowledge corroborate with the findings of Guirguis et al. [40]—the lower the age, the higher the number of NPS they were aware of. Given that most of the available educational resources on NPS are accessed online (e.g., NEPTUNE clinical guidelines or RIDR for reporting NPS adverse events), perhaps older HCPs may have less exposure to NPS-related information given their reduced use of electronic platforms.

### 4.2. NPS Types

Prescribed medications zopiclone, gabapentin and pregabalin were the most commonly known compounds listed in our survey, perhaps not unexpectedly. Feedback from the GP Shared Care CPD Event addressed a recurring theme of uncertainty about the misuse potential and proper use of medications like fentanyl and gabapentinoids. The analyses of databases provided by the EMA identified zopiclone as the most frequently reported ‘Z-drug’ involved in overdose and ADRs [55]. The findings also suggest caution be exercised when prescribing ‘Z-drugs’ to patients with psychiatric illness and/or history of drug abuse [55]. Moreover, benzodiazepines can enhance the effect of co-consumed opioids, but such co-use increases the risk of overdose through respiratory depression [33,56]. Similarly, pregabalin has been reported to cause central nervous system (CNS) depression when misused in combination with sedatives such as benzodiazepines, alcohol and opiates/opioids [57,58,59]. Our results suggest a high awareness of the NSO carfentanil; yet, most respondents were unaware of its misuse potential (Figure 5). The Global Drug Survey (2018) [60] stated that new opioids including acetyl fentanyl and carfentanil have caused numerous fatalities in the UK, Canada and the United States, thus requiring more awareness and greater vigilance [61]. Consequently, as a public health response, the NEPTUNE clinical guidelines about the harms and clinical management of NSOs were issued [62]. 

Amongst the SC, respondents were most aware of mephedrone. From the misuse of traditional substances such as ecstasy and cocaine, a shift towards injecting mephedrone is known to be popular in the ‘chemsex’ scene involving Men having sex with Men [63,64]. Among the miscellaneous NPS that were categorised as ‘other’, methoxetamine (*n* = 17; 13.3%) and PMA/PMMA (*n* = 15; 12.0%) were commonly known, whereas PMA/PMMA was sold as ecstasy in ‘superman’ tablets, which caused fatalities of party-goers on Christmas Eve 2015 [65]. Clearly, purity of sold substances is compromised where contamination often occurs with one or more controlled and uncontrolled substances [66]. Additionally, our data highlighted a poor awareness of SCRAs (e.g., MDMB-CHMICA, MDMB-FUBINACA) compared to other NPS groups (Figure 5). This is concerning as SCRAs are the largest group of NPS monitored by the EMCDDA [18]. Nonetheless, the ‘spice’ brand was frequently stated in the survey when asked to list what NPS were known to them. Using the street name ‘spice’ is inaccurate as it has become a generic term for SCRAs. Most NPS cannot be fully quantified where ingredients listed on the package are generally incomplete or false, with high variability between packages [67,68]. Further ingredients within ‘spice’ have been known to include the β2-mimetic clenbuterol, which potentially causes tachycardia and hypokalaemia, and tocopherol (vitamin E) possibly as a vehicle to improve intake via vaping [69]. 

### 4.3. Educational Sessions’ Feedback

Most HCPs expressed their need to learn more about NPS products, clinical warnings associated with them and their legal status. Although they expressed that their current source of knowledge is via peer support, media-style videos, websites, direct emails and scientific literature, they expressed their preference for online-based platforms on NPS updates, including emails, websites and online courses. However, resources must be cost-effective, regularly updated and widen the participation of most HCPs, including those who are not confident with electronic platforms. Furthermore, HCPs are often overloaded by various online training opportunities to aid their professional development. Attending a conference in which accreditation points can be collected would seem more feasible to improve their knowledge. Likewise, sending monthly newsletters to GP practices or having talks by specialists on NPS may cater to these HCPs. Overall, lectures, online and workshops were the most preferred methods of training. The World Health Organization (WHO) has recommended some effective educational strategies for HCPs [70]. These include targeted educational undergraduate and postgraduate programmes, the use of practical approaches such as artificial intelligence and point-of-care testing, evaluating effectiveness of educational methods, using indicators to monitor outcomes, developing trainers and educational resources and widening the range of HCPs being trained [70]. Further training provision for HCPs on NPS and the establishment of knowledge platforms for clinicians, health workers and social workers both locally and nationally is required [49]. This would enable the rapid dissemination of information about emerging NPS and the appropriate responses on the harms associated with them. Equally, implementation of multidisciplinary approaches by health providers in different settings (including sexual health clinics, custodial settings and drug treatment centres) can potentially improve referral pathways for NPS service users [71]. To our knowledge, evaluation of educational pedagogies for NPS have not been undertaken to date. 

### 4.4. Conceptualisation of an Evidence-Informed Public Health Approach to NPS use in the UK

Having an evidenced-based public health approach is fundamental to tackling NPS use. Uncertainties associated with the current and future public health burden of NPS suggests the importance of synthesising available data to inform national and international responses including interventions [72]. Certain NPS have transient life cycles, as 60 NPS seem to have disappeared from the market since 2013, whereas over 80 NPS became established on the global market between 2009 and 2015 [73]. Therefore, it may be inappropriate to employ evidence-based responses and regulation similar to those used for alcohol, tobacco and illicit drugs, due to the complex life cycles of NPS, along with the limited information available on their pharmacology and toxicology [74,75,76]. Challenges in identification and regulation are not only problematic for the legal system but also to HCPs who encounter NPS users. HCPs should have adequate knowledge about NPS classifications, user groups, NPS symptoms/effects and harm reduction strategies. In most cases, psychosocial interventions are employed in order to influence positive behavioural changes [77]. Front-line HCPs should be familiar with the PHE (Public Health England)/MHRA (Medicines and Healthcare products Regulatory Agency) initiatives such as RIDR and the MHRA Yellow Card Scheme, which allows HCPs to report cases of suspected and actual harm from illicit substance use and ADRs from medications, respectively. HCPs can refer to the NEPTUNE clinical guidance [27,62], along with online e-learning modules, which cover the clinical management of acute and chronic harm from NPS. Similarly, a quick reference guide and factsheet on NPS [51,78,79] and a section on the PSA (2016) can be found within one of the undergraduate and postgraduate essential resources for pharmacists and pharmacy students “*RPS Medicines, Ethics and Practice (MEP)*” [69], all of which have been co-developed by the corresponding author of this manuscript and are accessible via the RPS website for pharmacy professionals [79]. 

### 4.5. Implications and Recommendations for Policy-making and Clinical Practice

Specific treatment recommendations for ADRs associated with NPS is limited. Hence, PHE’s preliminary advice is to focus on individuals’ presenting symptoms rather than substance identification [43,77]. With the continuous growth of this market and increasing research, it became clear that identification of NPS through the classification of subcategories/subfamilies/derivatives with similar substructure or chemical backbone may assist HCPs in tailoring appropriate treatment for a patient [74]. In addition, chemical analyses of NPS voluntarily submitted by service users suggest that NPS were frequently consumed with other substances rather than in isolation [79]. In the era of digital healthcare, drug checking within a substance misuse service has been trialled by pharmacists and has enabled the provision of tailored harm reduction advice on the basis of a holistic multi-disciplinary approach [80]. Combining NPS with other substances may be intentionally used as a means to self-medicate to counteract side-effects of illicit substances [81]. However, users may unintentionally be consuming NPS mixtures with unclaimed ingredients [66]. Adulterants and bulking agents including benzocaine, caffeine, lidocaine, phenacetin and procaine were found to be commonly combined with NPS, especially in SC mixtures [81,82,83]. Their presence may produce synergistic, additive, supra-additive effects, and hence potentiate the effects of controlled drugs of abuse [84]. Similarly, involuntary contamination or presence of by-products from incomplete chemical reactions during the illicit manufacture of NPS may lead to inadvertent intoxication [85]. 

It is therefore recommended that GPs and non-medical prescribers should be vigilant when reviewing vulnerable individuals, especially those that are prescribed medicines that are particularly liable to misuse: gabapentinoids, antidepressants, antipsychotics and opioid analgesics [57]. Community pharmacists in primary care can provide harm reduction services including needle and syringe exchange/provision and advice on safer injecting techniques [79]. Additionally, during medicine reconciliations, NPS use may be identified by pharmacists, which may prompt appropriate advice to be given and/or referrals to be made [86]. Schifano and Chiappini [87] identified 1085 cardiovascular-related loperamide adverse drug reactions from the EMA’s EV during 2005-2017. Given that community pharmacists are potentially the first point-of-contact able to identify a repeat supply issue, their links with prescribers/clinicians should be enhanced for appropriate referrals to be made, thus allowing individuals to have more access to treatment services [88]. 

HCPs in hospitals, particularly nurses, pharmacists and doctors, should receive educational resources that are tailored to the clinical management of NPS-related adverse acute toxicity. Other HCPs including paramedics, first-responders, ambulance staff and first-aid responders should also access optimal and up-to-date NPS-related information about managing suspected toxicity arising from NPS use from the National Poisons Information Service (NPIS) through its telephone service and TOXBASE database. Care bundles can help in the management of acute intoxication and overdose (e.g., NEPTUNE care bundle on the harms of synthetic cannabinoid receptor agonists), particularly where individuals may have taken more than one substance [89,90]. Hence, treatment can be delivered safely and consistently within routine clinical practice. Similarly, the ‘Drugs Wheel’ designed by Mark Adley [91] can be utilised by HCPs, as it provides a simpler method of learning drugs by their category. 

Referrals to sexual health services can help educate individuals about the repercussions of partaking in high-risk sexual activities (e.g., ‘chemsex’) and address the prevention and treatment of sexually transmitted infections. Equally, it is also important to highlight the issue of emerging styles of alcohol consumption such as ‘binge’ drinking in future educational sessions for HCPs, as this has been reported by various studies as concurrent with poly-NPS abuse, which can potentially worsen a patient’s clinical presentation [92,93,94]. 

The private sectors providing drug treatment services should have access to evidence-based psychosocial and pharmacological approaches in order to increase an individual’s likelihood of recovery, as recommended by the ‘Orange Guidelines’ [95]. Ralphs and Gray [54] also recommended clearer referral pathways and strengthened links between existing drug treatment services, where more innovative strategies should be implemented to increase the engagement from NPS service users. 

Perhaps it may be more beneficial to provide objective regular assessments for HCPs, which test their awareness of NPS (e.g., clinical management, presenting symptoms, NPS classification). Wood et al. [43] recommended the utilisation of objective measures through simulation studies or objective structured clinical examinations (OSCEs) to ascertain knowledge. Potentially, this provides a more detailed measure of a HCP’s areas of strength or weakness and whether this is determined by how much exposure to NPS they obtain in their workplace. 

Lastly, databases, such as the EMA’s EV, which is considered worldwide as an exemplar, should be maintained to capture NPS-related adverse effects along with data about accident and emergency admissions [96]. Moreover, information-sharing should be improved at national, European and global levels to enable more prompt responses when highly toxic NPS are identified. 

### 4.6. Limitations of Research and Suggestions for Future Research

Purposive sampling via the distribution of the paper-based surveys to the respondents present in the events described above may have introduced a possible bias because it only gathered the views of a limited number of HCPs who may have genuine interest in knowing more about NPS. Additionally, it may be open to selection bias due to lack of random sampling [97]. Likewise, the Qualtrics survey may be limited to those who were only confident in answering the survey online. Given the cross-sectional nature of the survey, generalisability of our results should be treated with caution given that we have made inferences from a group of purposively sampled HCPs at one point in time. Indeed, our results provide a perception of their current baseline knowledge, but it should be emphasised that NPS consumption fluctuates regionally and over time [98]. Future studies should involve more data from these geographical regions as well as from Wales, Scotland and Northern Ireland and should include more representation of specialist doctors (e.g., general and addiction psychiatrists) and emergency service personnel (e.g., paramedics) to obtain an even distribution of HCPs, including private sectors, across the UK and in different settings, and to make the data more generalisable across the UK. Further research is needed in order to evaluate the pedagogic strategies employed to improve HCP’s knowledge of NPS. 

## 5. Conclusions

Despite the overall evidence of lack/poor knowledge of HCPs of NPS, the provision of educational CPD workshops showed an improvement within the self-reported knowledge of HCPs on NPS in comparison to previous studies. The health sector may have a considerable impact on NPS knowledge that HCPs can acquire. 

Continual education about NPS is indeed fundamental for the provision of improved harm reduction interventions, better referral pathways, effective dissemination of information and sharing of best practice to help NPS service users achieve full recovery and enhance their care throughout their treatment journey. Through this research, we recommend adopting innovative approaches for drug identification such as novel point-of-care testing that can be undertaken on-site, especially at emergency settings, by HCPs. We also recommend inclusion of integrated NPS toxicology/pharmacology/psychopathology education in undergraduate and postgraduate national healthcare curricula. The provision of regular updates through accredited quality-assured sector- and specialty-specific online platforms coupled with targeted and tailored educational tools. Connecting educators through peer-support to enhance the impact of these educational resources is also recommended. This is especially important, as NPS of varying types continue to emerge, and drug scenarios are ever-changing.

## Figures and Tables

**Table 1:** Comparison of type of practitioner by aspects of NPS use between health sectors.

Positive Response: Number Out of Total (%)					
	Hospital	Primary Care	Substance Misuse Service	Others	*p*-Value
Good/very good level of knowledge	11/77 (14.3)	8/68 (11.8)	8/28 (28.6)	4/7 (57.1)	0.008 *
Provide advice/counselling	40/72 (55.6)	20/55 (36.4)	19/26 (73.1)	4/4 (100.0)	0.002 *
Fairly/very confident about providing advice	34/77 (44.2)	21/68 (30.9)	15/28 (53.6)	5/7 (71.4)	0.051 *
Encounter customers/patients seeking advice	22/76 (28.9)	8/68 (11.8)	13/28 (46.4)	5/7 (71.4)	< 0.001
Fairly/very confident about providing advice on adverse effects	17/72 (23.6)	11/63 (17.5)	14/28 (50.0)	4/7 (57.1)	0.003
Knowledge on NPS/legal highs fairly/very significant in work	58/68 (85.3)	40/57 (70.2)	24/26 (92.3)	5/6 (83.3)	0.058 *

* Exact *p*-value.

**Table 2:** Comparison of type of practitioner by aspects of NPS use between professions.

Positive Response: Number Out of Total (%)					
	GP	Nurse	Pharmacist	Others	*p*-Value
Good/very good level of knowledge	0/17 (0.0)	10/50 (20.0)	16/106 (15.1)	4/12 (33.3)	0.086 *
Provide advice/counselling	8/16 (50.0)	38/48 (79.2)	31/90 (34.4)	5/6 (83.3)	<0.001 *
Fairly/very confident about providing advice	4/17 (23.5)	28/50 (56.0)	35/105 (33.3)	6/12 (50.0)	0.021
Encounter customers/patients seeking advice	5/17 (29.4)	21/50 (42.0)	19/104 (18.3)	4/12 (33.3)	0.018 *
Fairly/very confident about providing advice on adverse effects	3/16 (18.8)	21/49 (42.9)	18/97 (18.6)	2/10 (20.0)	0.013 *
Knowledge on NPS/legal highs fairly/very significant in work	10/17 (58.8)	41/46 (89.1)	69/87 (79.3)	8/9 (88.9)	0.052 *

* Exact *p*-value.

**Table 3:** Free text responses regarding other comments and suggestions.

Participant	Response
Nurse, mental health hospital	“It is an issue that still has elements of being unclear as what is legal and what is now illigel [sic], however the increase and potency of the drug on the street makes it an issue that affects all aspects of society.”
Pharmacist, substance misuse service	“NPS are not going away—and if anything, are going to get more designer and specific—we need to have understanding of their basics so we can formulate a base line approach as names and formulas change.”
General practitioner, general practice	“should be taught in schools about dangers of these. Use in universities seems very high”
Family safeguard worker, substance misuse service	“Please implement training as soon as possible. It is much needed to keep abreast of information and dangers on current legal highs.”
Pharmacist, community	“We have a significant problem with customers wanting to purchase codeine products in excess. These people are addicted to it but the only place to refer them to is the RISE addiction team who are overworked already.”

**Table 4:** Free text responses regarding learning from the GP Shared Care Continuing Professional Development (CPD) Event.

Participant	Response
GP 1	“Abuse of medications I didn’t realise had abuse potential; pharmacokinetics of Gaba meds/opiates & withdrawal of Benzos.”
GP 2	“misuse of prescriber drugs such as tramadol & fentanyl patch.”
GP 3	“Pregabalin/Gabapentin have high abuse potential.”
GP 4	“Massive growth in NPS. Actually feel helpless against this! I ran an EMIS search immediately. We have 5 ppl (out of 14000) on repeat for fentanyl patches. I’ll write to all regarding safe disposal.”
GP 5	“The pharmacodynamic pathways were really well explained & all makes a lot more sense again!; The number of potency of NPS’s -very scary.; The prevalence of NPS in herbal/food supps.”
GP 6	“Lots of things about NPS; RIDR; how various drugs are abused and dangers of withdrawal.”
GP 7	“The pharmacodynamic pathways were really well explained & it all makes a lot more sense again!”

**Table 5:** Free text responses regarding learning from the CPD Pharmacy Event.

Participant	Response
Ph 1	“Naloxone isn’t always suitable antagonist for NPS; Drug wars and NPS are very prevalent; Drug tests miss a lot of drugs of abuse…”
Ph 2	“Different classes of NPS; New substances being introduced all the time-not always detected; Rx drugs misused—gabapentin, pregabalin, venlafaxine.”
Ph 3	“How to educate patients on NPS; Different types of NPS; Potential drugs that can be abused (e.g., POMs).”
Ph 5	“NPS market, NPS user groups; What can we do? Harm reduction, awareness …”
Ph 6	“What NPS drugs; How dangerous NPS drugs are and the harm they can cause; Didn’t know before the presentation: Buscopan, Gabapentin, Pregabalin can be abused …”
Ph 8	“Challenges re using NPS-adverse reactions/toxicity/fatalities; Scale of the problem—in terms of use/difficulty monitoring; How quickly NPS are changing/its limitations in research …”

**Table 6:** Free text responses regarding future sessions and additional comments in the evaluation of GP Shared Care Event and CPD Pharmacy Event.

Participant	Response
Ph 4	“more updates….. on new drugs of abuse both legal POMs Ps etc… and illicit”
Ph 9	“Extremely informative & interesting session”
Ph 10	“Regular talks to update us on them!”
Ph 20	“To have more trainings, talks, workshops & to include also recovery workers; perhaps an NPS representative/head?”
GP 8	“Discussion/evidence on why people abuse meds. Discussion about…… drug addiction”
GP 9	“update on alcohol abuse”
GP 10	“Would be nice to have back to basic update + local services again some point”
GP 11	“How to identify and treat drug overdose /withdrawal.”

## Supplementary Materials

The following are available online at https://www.mdpi.com/2076-3425/10/3/142/s1, Table S1: NPS compounds; File S2: Survey instrument.
