# Supplementary material for: Exploring the Baseline Knowledge and Experience of Healthcare Professionals in the United Kingdom on Novel Psychoactive Substances
Source: Brain Sci. 2020 Mar 2;10(3):142. doi: 10.3390/brainsci10030142 (PMC7139605; doi:10.3390/brainsci10030142)
Supplement: Supplementary file 1 [file brainsci-10-00142-s001.pdf]

Supplementary Information 1. Have you heard any of the following drugs being abused?

	Yes	No	Know of patients/customers who have abused it	Total
Bupropion	70 (47.0 %)	72 (48.3 %)	7 (4.7 %)	149
Diphenhydramine	90 (57.7 %)	45 (28.8 %)	21 (13.5 %)	156
Gabapentin	106 (62.7 %)	22 (13.0 %)	41 (24.3 %)	169
Hyoscine butylbromide	64 (41.3 %)	81 (52.3 %)	10 (6.5 %)	155
Loperamide	71 (46.4 %)	64 (41.8 %)	18 (11.8 %)	153
Methylphenidate derivatives	92 (58.6 %)	47 (29.9 %)	18 (11.5 %)	157
Modafinil and Related products	72 (47.1 %)	68 (44.4 %)	13 (8.5 %)	153
Olanzapine	55 (36.9 %)	79 (53.0 %)	15 (10.1 %)	149
Orphenadrine	60 (40.8 %)	77 (52.4 %)	10 (6.8 %)	147
Promethazine	87 (55.4 %)	41 (26.1 %)	29 (18.5 %)	157
Pregabalin	103 (60.2 %)	20 (11.7 %)	48 (28.1 %)	171
Quetiapine	68 (43.9 %)	71 (45.8 %)	16 (10.3 %)	155
Sertraline	47 (32.0 %)	89 (60.5 %)	11 (7.5 %)	147
Tropicamide	11 (8.1 %)	123 (91.1 %)	1 (0.7 %)	135
Venlafaxine	45 (30.6 %)	94 (63.9 %)	8 (5.4 %)	147
Zopiclone	109 (64.1 %)	14 (8.2 %)	47 (27.6 %)	170
3-fluorofentanyl	31 (22.6 %)	100 (73.0 %)	6 (4.4 %)	137
3-phenylpropanoyl fentanyl	28 (20.6 %)	105 (77.2 %)	3 (2.2 %)	136
4Cl-iBF	8 (5.9 %)	127 (94.1 %)	N/A	135
4F-iBF	8 (6.0 %)	125 (94.0 %)	N/A	133
Acrylfentanyl (Acryloylfentanyl)	18 (15.1 %)	100 (84.0 %)	1 (0.8 %)	119
AH-7921	5 (3.8 %)	128 (96.2 %)	N/A	133
Benzoyl fentanyl	39 (28.3 %)	96 (69.6 %)	3 (2.2 %)	138
Carfentanil	41 (29.3 %)	98 (70.0 %)	1 (0.7 %)	140
Cyclopropyl fentanyl	28 (20.4 %)	108 (78.8 %)	1 (0.7 %)	137
Furanyl fentanyl	32 (23.4 %)	104 (75.9 %)	1 (0.7 %)	137
MT-45	8 (6.2 %)	120 (93.0 %)	1 (0.8 %)	129
Tetramethylcyclopropane-fentanyl	16 (12.1 %)	115 (87.1 %)	1 (0.8 %)	132
THF-F	6 (4.7 %)	122 (94.6 %)	1 (0.8 %)	129
U-47700	2 (1.5 %)	127 (97.7 %)	1 (0.8 %)	130
W18	3 (2.3 %)	126 (96.9 %)	1 (0.8 %)	130
W-45	2 (1.6 %)	126 (97.7 %)	1 (0.8 %)	129
4-Chlorodiazepam	46 (32.9 %)	88 (62.9 %)	6 (4.3 %)	140
Bromazolam	18 (13.2 %)	116 (85.3 %)	2 (1.5 %)	136
Etizolam	16 (11.9 %)	118 (87.4 %)	1 (0.7 %)	135
Flubromazepam	15 (10.9 %)	123 (89.1 %)	N/A	138
Flubromazolam	14 (10.4 %)	121 (89.6 %)	N/A	135
Pyrazolam	14 (10.3 %)	122 (89.7 %)	N/A	136
Phenazepam	19 (13.8 %)	118 (85.5 %)	1 (0.7 %)	138
Ro 07-4065	4 (3.1 %)	123 (96.9 %)	N/A	127
5Cl-MDMB-PINACA	3 (2.3 %)	128 (97.7 %)	N/A	131
5F-3,5-AB-PFUPPYCA	2 (1.5 %)	130 (98.5 %)	N/A	132
5F-AMB	4 (3.0 %)	129 (97.0 %)	N/A	133
5F-MDMB-PINACA	4 (3.1 %)	127 (96.9 %)	N/A	131

5F-PB-22	3 (2.3 %)	129 (97.0 %)	1 (0.8 %)	133
5F-UR-144	3 (2.3 %)	128 (97.7 %)	N/A	131
AB-CHMINACA	3 (2.3 %)	128 (97.7 %)	N/A	131
AMB-FUBINACA	3 (2.3 %)	129 (97.7 %)	N/A	132
BB-22	3 (2.3 %)	129 (97.7 %)	N/A	132
Cumyl-4CN-B7AICA	3 (2.3 %)	129 (97.7 %)	N/A	132
Cumyl-4CN-BINACA	3 (2.3 %)	127 (97.7 %)	N/A	130
Cumyl-5F-PINACA	4 (3.1 %)	126 (96.9 %)	N/A	130
FUB-PB-22	3 (2.3 %)	127 (97.7 %)	N/A	130
MAM-2201	3 (2.3 %)	127 (96.9 %)	1 (0.8 %)	131
MDMB-CHMICA	6 (4.6 %)	125 (95.4 %)	N/A	131
MDMB-FUBINACA	6 (4.6 %)	125 (95.4 %)	N/A	131
MN-18	3 (2.3 %)	128 (97.7 %)	N/A	131
SDB-005	2 (1.5 %)	128 (97.7 %)	1 (0.8 %)	131
STS-135	2 (1.6 %)	126 (98.4 %)	N/A	128
THJ-2201	2 (1.6 %)	127 (98.4 %)	N/A	129
JWH-018	3 (2.3 %)	125 (97.7 %)	N/A	128
3-CMC	3 (2.4 %)	121 (96.8 %)	1 (0.8 %)	125
4-Chloro-alpha-PPP	2 (1.6 %)	122 (96.8 %)	2 (1.6 %)	126
4-Chloro-alpha-PVP	2 (1.6 %)	123 (96.9 %)	2 (1.6 %)	127
4-CMC	3 (2.4 %)	123 (96.9 %)	1 (0.8 %)	127
Alfa- PVP; Alfa -PPP	2 (1.6 %)	122 (97.6 %)	1 (0.8 %)	125
Alpha-PVT	4 (3.2 %)	121 (96.0 %)	1 (0.8 %)	126
Butylone (bk-MBDB, B1)	6 (4.8 %)	119 (94.4 %)	1 (0.8 %)	126
Dibutylone	10 (8.0 %)	114 (91.2 %)	1 (0.8 %)	125
Dimethylone	10 (7.8 %)	116 (90.6 %)	2 (1.6 %)	128
Ethylone	12 (9.4 %)	113 (88.3 %)	3 (2.3 %)	128
Flephedrone (4-FMC)	6 (4.8 %)	116 (93.5 %)	2 (1.6 %)	124
MDPV	8 (6.4 %)	116 (92.8 %)	1 (0.8 %)	125
Mephedrone (4-MMC, drone, meow)	62 (44.9 %)	61 (44.2 %)	15 (10.9 %)	138
Methylone	11 (8.6 %)	115 (89.8 %)	2 (1.6 %)	128
N-Ethylpentylone	6 (4.8 %)	117 (94.4 %)	1 (0.8 %)	124
Pentylone	8 (6.2 %)	118 (91.5 %)	3 (2.3 %)	129
TH-PVP	5 (4.0 %)	120 (95.2 %)	1 (0.8 %)	126
2C-C NBOMe	4 (3.1 %)	125 (96.9 %)	N/A	129
2-MeO-diphenidine	8 (6.3 %)	119 (93.7 %)	N/A	127
3- & 4-MeO-PCP	9 (7.1 %)	116 (92.1 %)	1 (0.8 %)	126
4,4’-DMAR;)	9 (7.1 %)	117 (92.9 %)	N/A	126
5-MeO DMT (Alpha O)	10 (7.9 %)	114 (90.5 %)	2 (1.6 %)	126
5- & 6-APB (Benzofury)	12 (9.4 %)	112 (88.2 %)	3 (2.4 %)	127
25I-NBOMe	8 (6.3 %)	118 (93.7 %)	N/A	126
BZP (Benzylpiperazine)	14 (11.0 %)	110 (86.6 %)	3 (2.4 %)	127
Diphenidine	16 (12.5 %)	111 (86.7 %)	1 (0.8 %)	128
DNP (2,4-Dinitrophenol)	11 (8.7 %)	113 (89.7 %)	2 (1.6 %)	126
Kratom (mitragynine speciosa)	8 (6.3 %)	116 (92.1 %)	2 (1.6 %)	126
MCPP	9 (7.1 %)	117 (92.9 %)	N/A	126
MEAI (Chaperon)	3 (2.4 %)	121 (96.8 %)	1 (0.8 %)	125
Methoxetamine	17 (13.3 %)	109 (85.2 %)	2 (1.6 %)	128
PMA; PMMA	15 (12.0 %)	110 (88.0 %)	N/A	125
